# Unraveling the characteristics of microRNA regulation in the developmental and aging process of the human brain

**DOI:** 10.1186/1755-8794-6-55

**Published:** 2013-12-09

**Authors:** Weiguo Li, Lina Chen, Wan Li, Xiaoli Qu, Weiming He, Yuehan He, Chenchen Feng, Xu Jia, Yanyan Zhou, Junjie Lv, Binhua Liang, Binbin Chen, Jing Jiang

**Affiliations:** 1 College of Bioinformatics Science and Technology, Harbin Medical University, Harbin, Heilongjiang Province, China; 2 Institute of Opto-electronics, Harbin Institute of Technology, Harbin, Heilongjiang Province, China; 3 National Microbology Laboratory, Public Health Agency of Canada, Winnipeg, MB, Canada

**Keywords:** Human brain, Development, Aging, miRNA, Synergistic regulation

## Abstract

**Background:**

Structure and function of the human brain are subjected to dramatic changes during its development and aging. Studies have demonstrated that microRNAs (miRNAs) play an important role in the regulation of brain development and have a significant impact on brain aging and neurodegeneration. However, the underling molecular mechanisms are not well understood. In general, development and aging are conventionally studied separately, which may not completely address the physiological mechanism over the entire lifespan. Thus, we study the regulatory effect between miRNAs and mRNAs in the developmental and aging process of the human brain by integrating miRNA and mRNA expression profiles throughout the lifetime.

**Methods:**

In this study, we integrated miRNA and mRNA expression profiles in the human brain across lifespan from the network perspective. First, we chose the age-related miRNAs by polynomial regression models. Second, we constructed the bipartite miRNA-mRNA regulatory network by pair-wise correlation coefficient analysis between miRNA and mRNA expression profiles. At last, we constructed the miRNA-miRNA synergistic network from the miRNA-mRNA network, considering not only the enrichment of target genes but also GO function enrichment of co-regulated target genes.

**Results:**

We found that the average degree of age-related miRNAs was significantly higher than that of non age-related miRNAs in the miRNA-mRNA regulatory network. The topological features between age-related and non age-related miRNAs were significantly different, and 34 reliable age-related miRNA synergistic modules were identified using Cfinder in the miRNA-miRNA synergistic network. The synergistic regulations of module genes were verified by reviewing miRNA target databases and previous studies.

**Conclusions:**

Age-related miRNAs play a more important role than non age-related mrRNAs in the developmental and aging process of the human brain. The age-related miRNAs have synergism, which tend to work together as small modules. These results may provide a new insight into the regulation of miRNAs in the developmental and aging process of the human brain.

## Background

Structure and function of the human brain change dynamically during its development and aging. The molecular and structural transformations, which form the human cognitive function, occur mainly in the period between birth and adulthood, and some developmental processes extend into adulthood, such as cortical axon myelinization [1-3]. The aging process of human brain begins at early adulthood. The aging-related changes include a decrease of brain volume, loss of synapses, and cognitive decline [2,4-6]. In later life, the brain starts to change in a more destructive manner, which leads to a continuous cognitive decline and a rise in the frequency of neurological disorders including Alzheimer’s disease and Parkinson’s disease [7-9]. Although the changes in the developmental and aging process of the human brain are clearly observed in histology and cognitive function, the underlining molecular mechanisms are not well understood.

MicroRNAs (miRNAs) are a class of small non-coding RNAs that regulate gene expression by promoting degradation or repressing translation of target mRNAs in post-transcriptional level. Moreover, some miRNAs have also been observed to activate transcription and translation of the targets [10,11]. Many studies have demonstrated that miRNAs play important roles in many biological functions and human diseases, such as cell proliferation, differentiation, development, apoptosis, neuronal development, differentiation, synaptic plasticity, and tumor development [12]. In the developmental process of the human brain, several lines of evidence indicated that miRNAs contribute to the control of the development, functional and structural reorganization of the human brain [13]. For example, neuron-specific miR-124 promotes neuronal differentiation by directly targeting PTB, which encodes a global repressor for alternative pre-mRNA splicing in non-neuronal cells [14]. MiR-134, which is localized to the synaptodendritic compartment of hippocampal neurons, regulates synaptic plasticity by inhibiting translation of Lim-domain–containing protein kinase 1 (LIMK1) [15]. Interestingly, accumulated evidence indicated that specific miRNAs have been shown to be involved in brain aging and other neurodegenerative pathologies [16-18]. miRNAs can regulate pathways involved in aging, and are significantly up- or down-regulated in their expression levels [19]. There are around 1100 miRNAs in the human genome [20,21], which potentially regulate the majority of all human genes [22]. Therefore, these miRNAs may guide many important biological processes ranging from proliferation, differentiation to senescence and apoptosis [23-25]. It has been shown that one miRNA could regulate hundreds of target genes [26]. Moreover, the limited miRNAs are able to regulate a large number of genes through synergism, in which multiple miRNAs work synergistically to regulate individual genes [27]. For example, Krek et al. [28] found that gene Mtpn was simultaneously regulated by miR-124, let-7b and miR-375, which is the positive evidence for cooperative miRNA control in mammals. Wu et al. [29] showed that 28 miRNAs could substantially inhibit the expression of p21Cip1/Waf1. Therefore, the regulations between miRNAs and predicted targets could be understood more comprehensively from the network perspective. The characteristic, that one miRNA regulates a larger number of genes and one target gene is jointly regulated by multiple miRNAs, implies a complex regulatory network between miRNAs and mRNAs. Studying this complex regulatory network and the synergism of miRNAs would provide new insights into the molecular basis of miRNA functions at a system level.

Traditionally, development and aging are studied separately, which may not completely interpret the physiological mechanism over the entire lifespan. It was recently found that the majority of miRNAs and gene expression changes occurring in aging represent reversals or extensions of developmental patterns [30]. Thus, it is necessary to study the regulatory effect between miRNAs and mRNAs in the developmental and aging process of the human brain.

**Figure 1:**
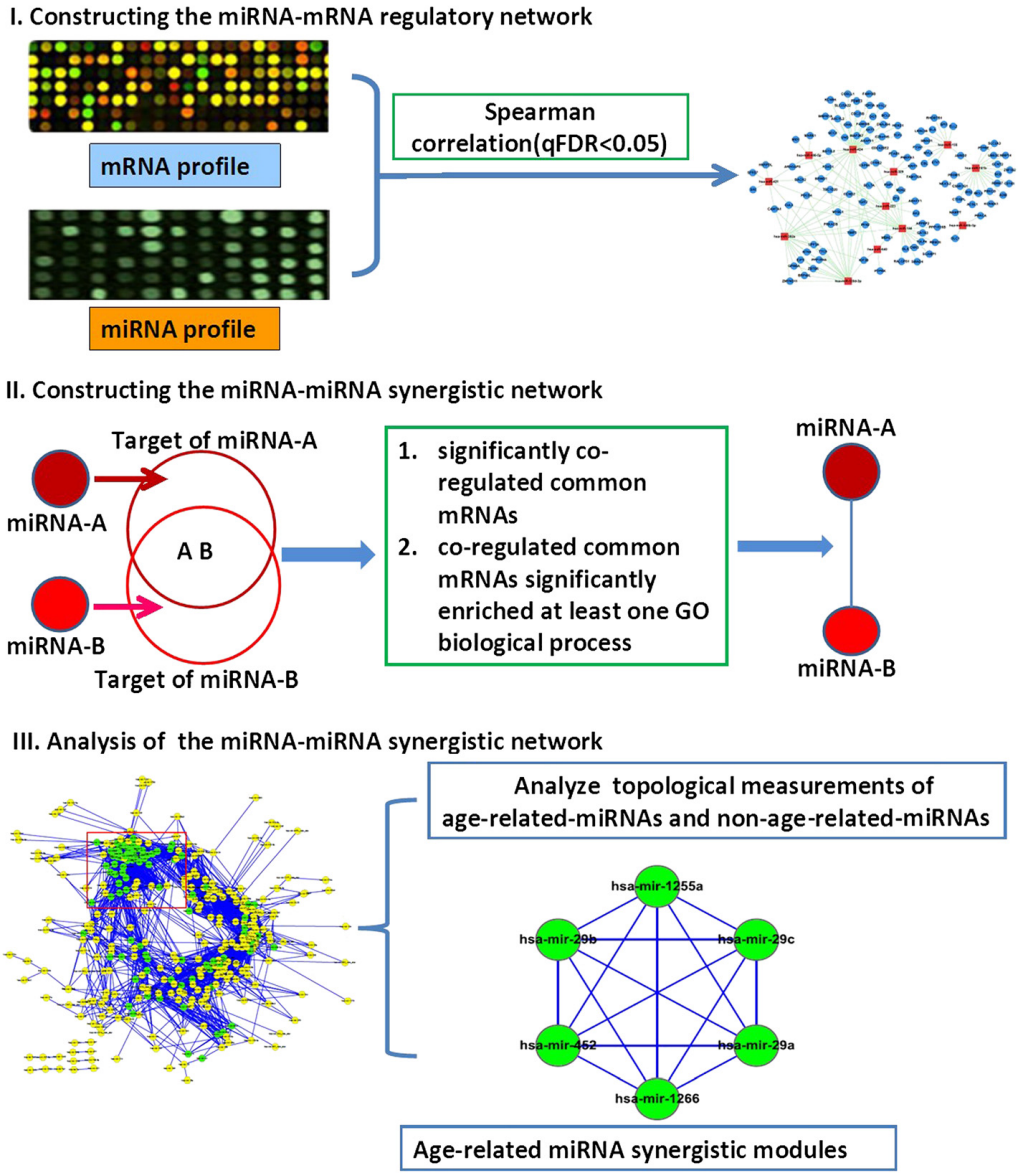
**The workflow of procedure. I)** The workflow to construct the miRNA-mRNA regulatory network. The bipartite miRNA-mRNA regulatory network was constructed by pair-wise correlation coefficient analysis between miRNA and mRNA expression profiles. **II)** The workflow to construct the miRNA-miRNA synergistic network. The miRNA-miRNA synergistic network was constructed based on co-regulation of target genes and GO function enrichment of co-regulated target genes. **III)** The analysis of the miRNA-miRNA synergistic network. We compared topological measurements between age-related-miRNAs and non age–related–miRNAs, and identified miRNA synergistic modules from the miRNA-miRNA synergistic network by Cfinder.

In this study, we first constructed a bipartite miRNA-mRNA regulatory network by analyzing pair-wise correlation coefficients between miRNA and mRNA expression profiles in the prefrontal cortex of humans throughout the lifetime. The miRNA-miRNA synergistic network was then built from the miRNA-mRNA network. The workflow of procedure is shown in Figure 1. The generated two networks will be explored to reveal the regulatory characteristics of miRNAs in the whole life of the human brain.

## Methods

### Data used in the study

The mRNA and miRNA expression data (GSE18069) in the prefrontal cortex of humans was downloaded from the GEO database [30]. It contains 23 cognitively healthy individuals with ages ranging from 2 days to 98 years old. The mRNA expression profile was measured using the Affymetrix Human Gene 1.0 ST platform and its normalized data set was downloaded. In the mRNA data, probe set identifiers (IDs) were mapped to ensemble gene IDs and mean expression level from multiple probe sets corresponding to the same gene was used to represent its expression level. The miRNA data was generated using Illumina high-throughput sequencing, which was derived from the analysis of the miRNA expression in 12 subjects selected from the individuals studied at the mRNA level. The abundance of miRNAs was normalized as RPM (reads per million reads) [31].

Candidate human miRNA–target relationships were acquired from miRNA target databases: TargetScan [32], miRanda [33], DIANA-microT [34], PicTar5 [28], RNAhybrid [35], RNA22 [36], PITA [37], MirTarget [38], TargetMiner [39] and mirSVR [40]. In order to improve the reliability of the predicted miRNA regulations, the regulations that were stored in at least three databases were extracted for our study.

### Selection of age-related miRNAs

To test the effect of age on miRNA expression level for selecting the age-related miRNAs, polynomial regression models were applied [30]. For each miRNA, the best regression model with the highest “adjusted r^2^” value from families of cubic polynomial regression models was selected [41]. More specifically, we fit a third degree regression model with age for each miRNA:

(1) $$y_{\mathrm{ij}}=b_{0i}+b_{1i}A_{j}+b_{2i}A_{j}^{2}+b_{3i}A_{j}^{3}+e_{\mathrm{ij}}$$

where *y*_*ij*_ is the expression level for gene *i* with *i* = 1, ⋯, *m* and sample *j* with *j* = 1, ⋯, *n*, *A*_*j*_ is the age of the sample *j*, and *e*_*ij*_ is the error term.

Then, we further calculated the six possible submodels of Equation (1), for example:

$$\begin{array}{ll} y_{\mathrm{ij}} & =b_{0i}+b_{1i}A_{j}+e_{\mathrm{ij}},y_{\mathrm{ij}}\\ & =b_{0i}+b_{1i}A_{j}+b_{2i}A_{j}^{2}+e_{\mathrm{ij}},\mathrm{etc}. \end{array}$$

Finally, we compared all seven models to the null model,

(2) $$y_{\mathrm{ij}}=b_{0i}+e_{\mathrm{ij,}}$$

by means of an F-test. We chose the model with the highest “adjusted r^2^” value as the best choice.

To generate the “age-related” miRNAs, the significance of the chosen regression model was estimated with the F-test, and the FDR was calculated by 1000 random permutations of age. The median of the permutation distribution was used as the null expectation. For the miRNA data set, miRNAs with an age-test FDR < 0.1% were defined as “age-related”. Analyses were conducted in the R environment. The R code used in the analyses can be found at http://www.picb.ac.cn/Comparative/data.html[42].

### Construction of the miRNA-mRNA regulatory network

MiRNAs can regulate mRNAs through binding to its 3′UTR, and can also regulate other miRNAs through indirect regulation. To comprehensively interpret the possible miRNA-mRNA regulatory effects at the whole genome scale, we constructed the miRNA-mRNA regulatory network by performing pair-wise spearman correlation coefficient analysis to evaluate potential correlations between 554 miRNA and 12,281 mRNA expression levels on 12 human brain samples. False discovery rate q value (qFDR), computed by the QVALUE software [43], was used to evaluate the statistical significance of miRNA-mRNA pairs. The miRNA-mRNA regulatory network was constructed by assembling all the significant miRNA-mRNA pairs (qFDR < 0.05), in which nodes represented miRNAs and mRNAs, and edges represented their potential regulatory correlations.

### Construction of the miRNA-miRNA synergistic network

According to the miRNA-mRNA regulatory network, we constructed miRNA–miRNA synergistic network based on enrichment analysis. In the miRNA–miRNA synergistic network, two miRNAs were connected if they significantly co-regulated common mRNAs, which were significantly enriched in at least one GO biological process term. The enrichment analysis was performed by cumulative hypergeometric distribution. The two connected miRNAs were considered to have synergistic relationships. The formula was as follows:

(3) $$E=\left\{E_{1}\left|\sum_{i_{1}=k_{1}}^{\min\left(n_{1},j_{1}\right)}\frac{\left(\begin{array}{c} j_{1}\\ i_{1} \end{array}\right)\left(\begin{array}{c} m_{1}-j_{1}\\ n_{1}-i_{1} \end{array}\right)}{\left(\begin{array}{c} m_{1}\\ n_{1} \end{array}\right)}<0.05\right)\right\}\cap \left\{E_{2}\left|\sum_{i_{2}=k_{2}}^{\min\left(n_{2},j_{2}\right)}\frac{\left(\begin{array}{c} j_{2}\\ i_{2} \end{array}\right)\left(\begin{array}{c} m_{2}-j_{2}\\ n_{2}-i_{2} \end{array}\right)}{\left(\begin{array}{c} m_{2}\\ n_{2} \end{array}\right)}\right)<0.05\right\}$$

The first part is the set of miRNA pairs, which significantly co-regulated mRNAs. Here, *k*_1_ is the number of mRNAs regulated by both miRNAs, *m*_1_ denotes the total number of mRNAs that were regulated by all miRNAs, *n*_1_ represents the number of mRNAs that were correlated with one miRNA, and *j*_1_ denotes the number of mRNAs that were correlated with the other miRNA. The second part is the set of miRNA pairs whose target mRNAs were enriched in a GO biological process. Here, *k*_2_ is the number of mRNAs included in GO terms, *m*_2_ is the number of mRNAs significantly co-regulated by miRNA pairs, *n*_2_ is the number of mRNAs that could not be annotated to any GO terms, and *j*_2_ is the number of mRNAs that are not significantly co-regulated by miRNA pairs and are also annotated to the GO terms.

### Topological measurements of network

For the two constructed networks, we analyzed several topological features. For the whole network, we examined the degree distribution of the network. The nodes degree distribution *N*(*k*) was defined to be the number of nodes with degree *k*. We also calculated degree, clustering coefficient and average shortest path length of nodes. The degree of a node is the number of edges linked to the node [44]. The average degree of nodes was the mean degree value of all nodes in a certain set. The shortest path is a path with the smallest number of links between two nodes. The average shortest path length of a node is the average length of the shortest paths between the node and any other nodes. For a given subset of nodes, we defined its characteristic path length as the average shortest path length between any two nodes of the set.

### Identification of age-related miRNA synergistic modules

We applied the Cfinder [45], a software based on the clique percolation clustering method, to identify miRNA synergistic modules from the miRNA-miRNA synergistic network. We defined modules as cliques, which are maximal complete subgraphs in the network. In each clique, every two miRNAs in the subgraph were connected by an edge.

For the purpose of selecting the age-related miRNA synergistic modules, we calculated the proportion of age-related miRNAs in modules and tested the correlation between the expression levels of the modules and age. The average expression of all miRNAs in a module was used to represent the overall expression level of the module. We used Pearson’s correlation coefficient to evaluate the correlation between the expression levels of the modules and age.

We evaluated the significance of the proportion of age-related miRNAs in modules and the correlation of the expression levels of the modules with age by randomly selecting miRNAs as miRNA modules. For each miRNA module, we randomly selected 1000 modules with the same number of miRNAs, calculated the proportion of age-related miRNAs in modules and evaluated the correlation between the expression levels of the modules and age. Modules with both the proportion and the correlation greater than the value in the real condition were recorded. The significance P-value was the fraction of these modules in 1000.

## Results

### Age-related-miRNAs

Using polynomial regression models, following Somel et al. [30] (see ‘Methods’ section), we found 98 age-related miRNAs (FDR < 0.001), whose expression levels showed significant changes with age.

### MiRNA-mRNA regulatory network

The preliminary miRNA-mRNA regulatory network was first constructed by performing pair-wise spearman correlation coefficient analysis between miRNA and mRNA expression profiles. In this network, we detected 36618 significantly correlated miRNA-mRNA pairs (qFDR < 0.05). These significant miRNA-mRNA pairs were then assembled to form the final miRNA-mRNA regulatory network. The resulted network consisted of 36618 regulations between 401 miRNAs and 7175 mRNAs which represented potential regulatory correlation between miRNAs and mRNAs at the whole genome-scale. 93.5% of the miRNAs regulated at least two mRNAs and 70.4% of mRNAs were co-regulated by over two miRNAs. These results demonstrated a complicated combination in terms of target-mRNA multiplicity.

**Figure 2:**
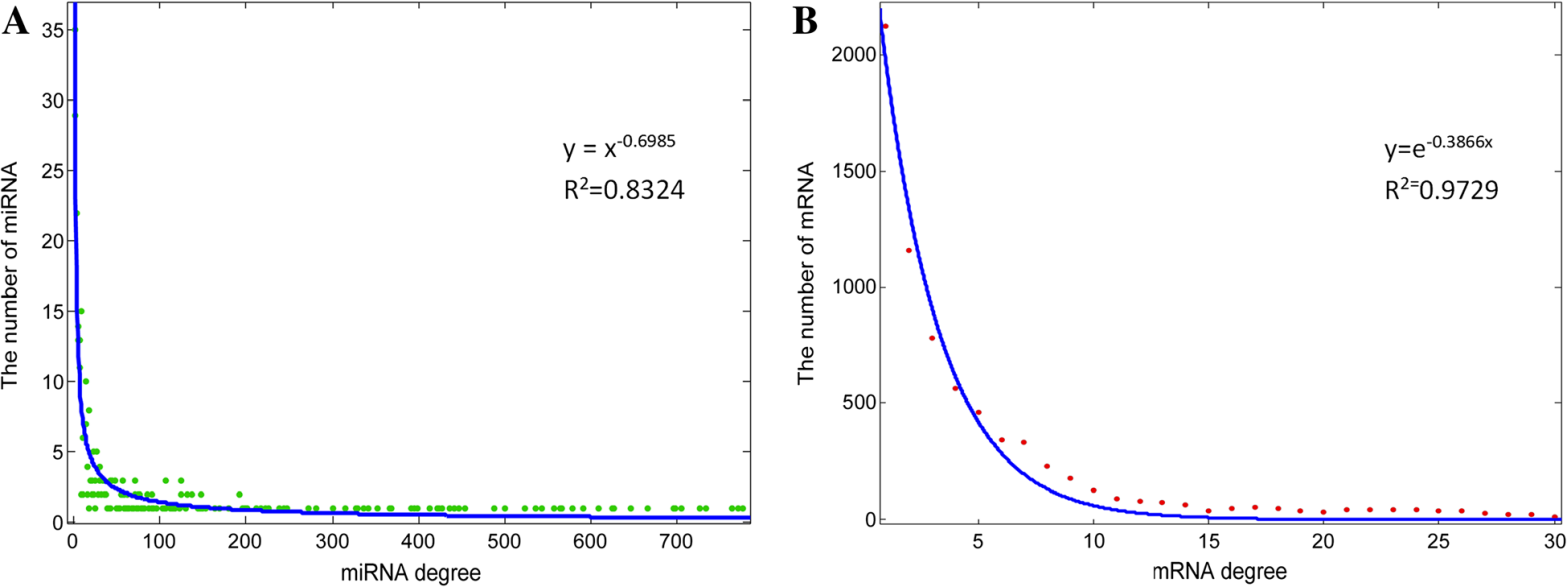
**Degree distribution of the miRNA-mRNA network. A)** out-degree distribution of the miRNA-mRNA network. Most of miRNAs are lowly connected and only a few are relatively highly connected. The examination of the out-degree distribution of the miRNA-mRNA network reveals a power law with a exponent of -0.6985 and R^2^ =0.8324. **B)** in-degree distribution of the miRNA-mRNA network. In-degree is defined as the number of regulatory miRNAs for each target, signifying an exponential distribution with an exponent of -0.3866 and R^2^ = 0.9729. The exponent described the distribution of degrees. R^2^ is the coefficient of determination, which indicates how well data points fit a line or curve. An R^2^ of 1 indicates that the line perfectly fits the data. The larger R^2^ is, the better the data fit the line.

We also examined the degree distribution of miRNAs and mRNAs in the network, and observed a power law and an exponential distribution, respectively (Figure 2). Moreover, the miRNA-mRNA network displayed scale-free characteristics, suggesting that the miRNA-mRNA network was characterized by a core set of organizing principles in its structure [46]. This feature implied that the miRNA-mRNA network accorded with general biological networks.

**Figure 3:**
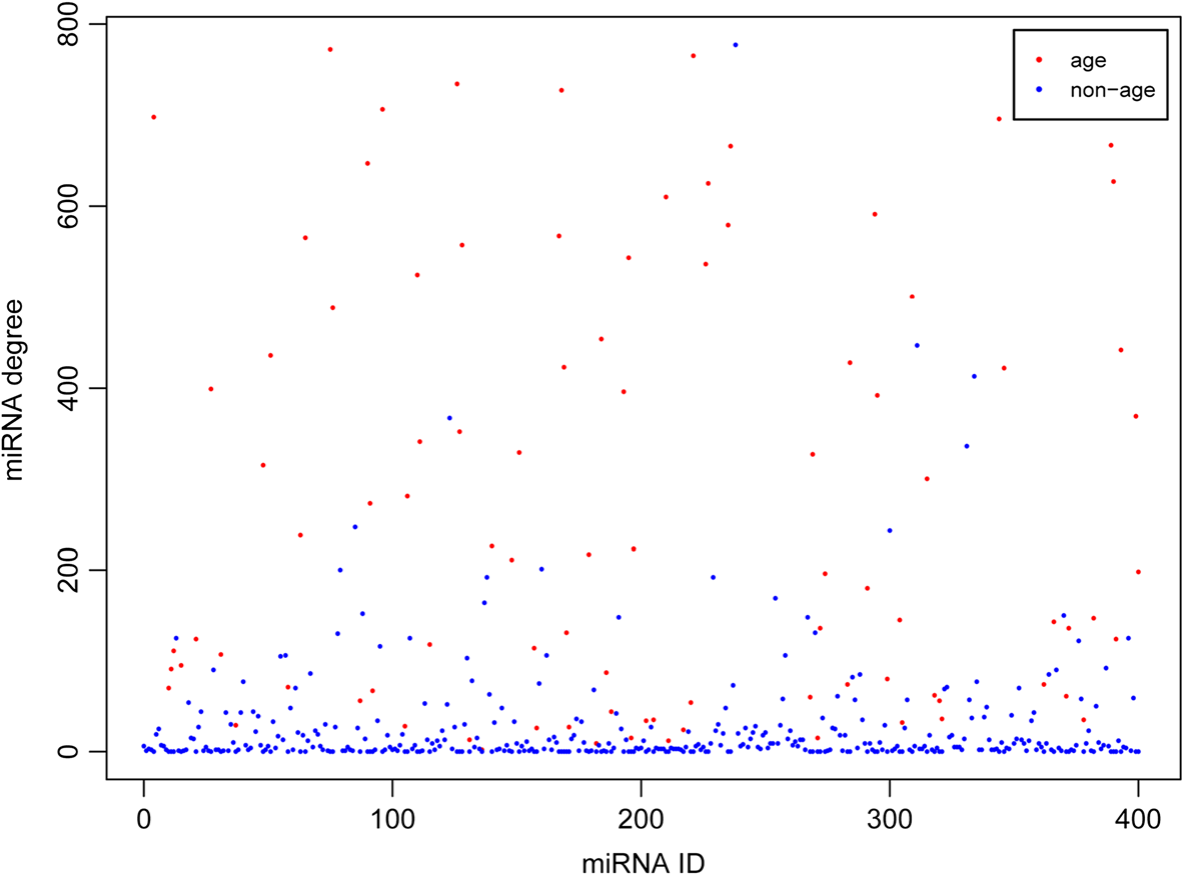
**Scatter plot of miRNAs degree distribution in the miRNA-mRNA network.** Y-axis represents miRNA degree, and x-axis is all miRNAs in the miRNA-mRNA network. Red and blue dots represent age-related miRNAs and non age-related miRNAs, respectively. Age-related miRNAs had higher degrees than non age-related miRNAs, which indicated that age-related miRNAs (*e.g.* hsa-mir-130a, has-mir-330-3p and hsa-mir-29a) regulated much more mRNAs than non age-related miRNAs in the miRNA-mRNA network.

The differences of degree distribution between age-related miRNAs and non age-related miRNAs were significant according to the Wilcoxon rank sum test (p < 2.2e-16) (Figure 3). The average degree of age-related miRNAs was 277.0753, whereas that of non age-related miRNAs was 35.22727. The result suggested that age-related miRNAs could regulate much more mRNAs than non age-related miRNAs.

**Figure 4:**
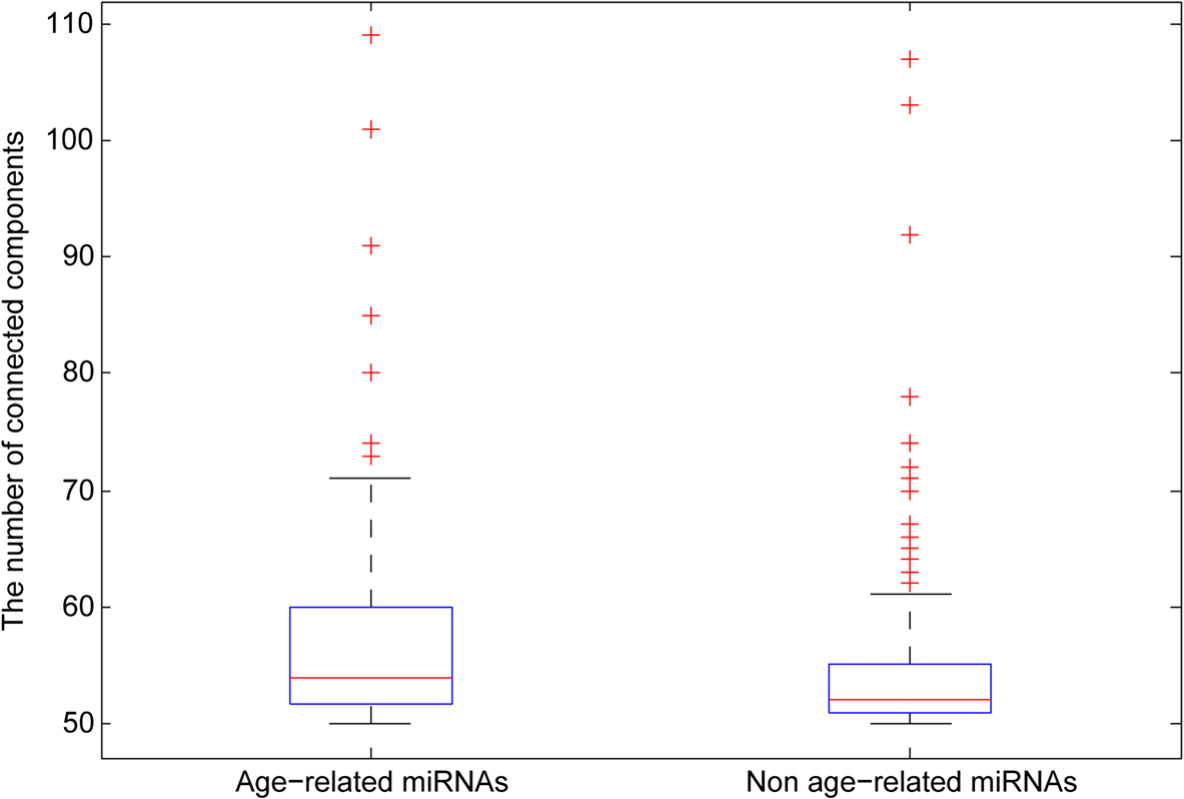
**The number of connected components compared for networks deleting age-related miRNAs and non age-related miRNAs.** The lower and upper lines of the boxes are the 25th and 75th percentiles of the measure. The lines in the middle of the boxes are the median. Lines extending above and below the boxes show the extent of the rest of the sample. The plus signs at the top and bottom of the figures are indications of outliers.

To further investigate whether age-related miRNAs played more important roles in the network, we deleted each age-related miRNA and non age-related miRNA from the miRNA-mRNA network, respectively, and compared the number of connected components in the remaining networks. By comparison, the measures of each remaining network after deleting the age-related miRNAs were significantly larger than those of non age-related ones (p < 2.2e-5) (Figure 4), which implied that age-related miRNAs connected and regulated more mRNAs in the miRNA-mRNA network. These results above indicated that age-related miRNAs played more important roles in the developmental and aging process of the human brain.

### MiRNA-miRNA synergistic network

**Figure 5:**
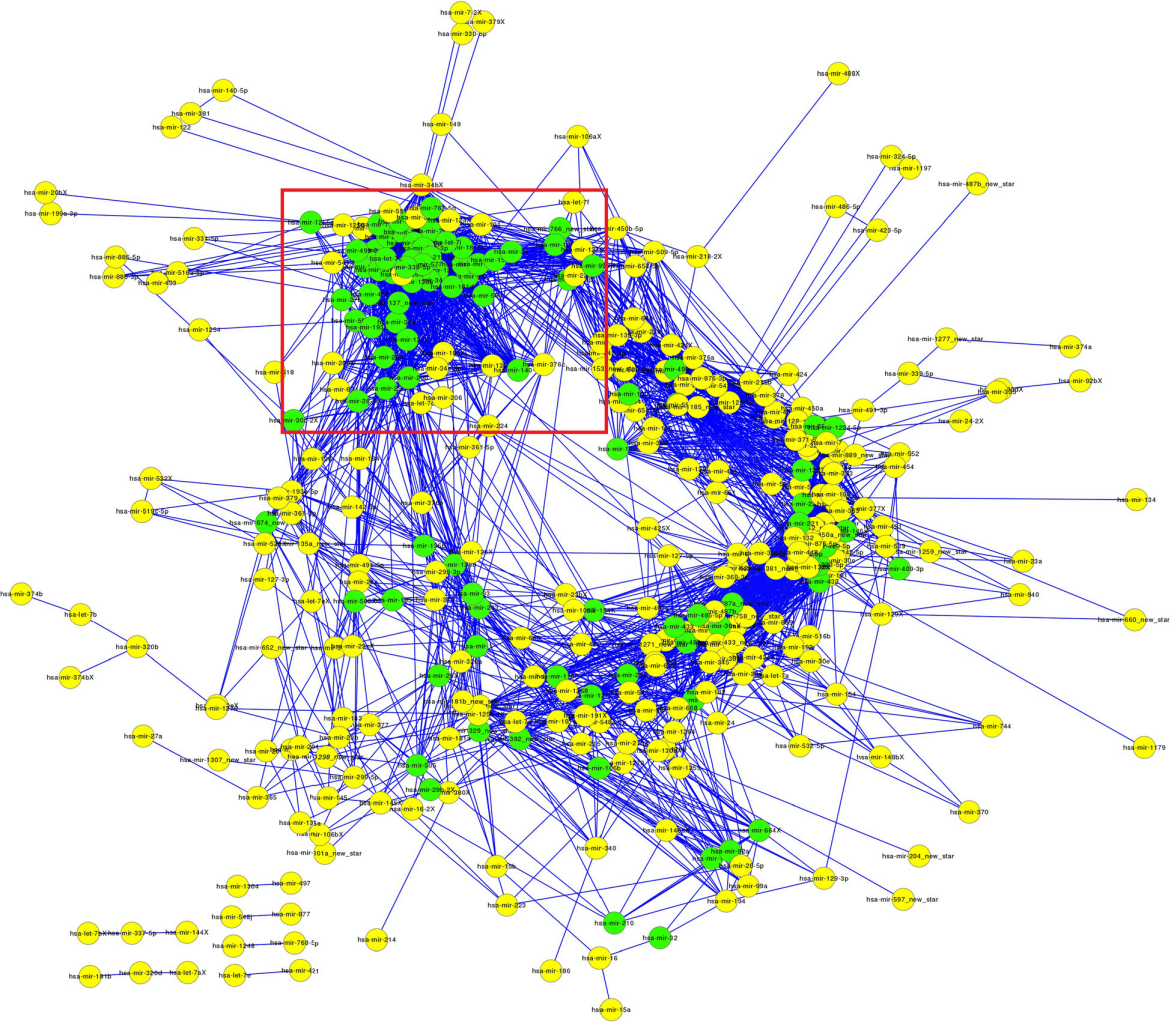
**The layout of the miRNA-miRNA synergistic network.** The miRNA-miRNA synergistic network generated by the procedure described in ‘Methods’. This network consists of 324 miRNAs and 3141 co-regulatory links. A node represents a miRNA, and an edge represents a synergistic action. Green and yellow circles represent age-related miRNAs and non age-related miRNAs, respectively. Age-related miRNAs are closer to each other in the red rectangle of the network.

Most mRNAs were co-regulated by over two miRNAs in the miRNA-mRNA network suggested the synergism of miRNA. To study the synergism of miRNAs at the system level in the developmental and aging process of the human brain, we constructed a miRNA-miRNA synergistic network from the miRNA-mRNA regulatory network. In the network, there were 324 miRNAs and 3141 edges (Figure 5). The connected miRNA pairs were shown to work synergistically through co-regulated mRNAs in special biological process. Furthermore, the miRNAs degree in the miRNA-miRNA synergistic network followed the power law distribution with a slope of -0.5386 and R^2^ = ~0.8153 and displayed scale-free characteristics.

To further evaluate synergy of miRNAs in the network, we generated random miRNA-miRNA synergistic network by keeping the degree of each node unchanged using the ‘RandomNetworks’ plugin of Cytoscape. The clustering coefficients of the random network were significantly smaller than those of the actual network (p < 2.2e-16). The average clustering coefficient of the actual network was 0.5221 compared to 0.1605 of the random network, suggesting the dense local neighborhoods of the actual network. The immediate neighbors of a miRNA tend to be synergistic, which are functional synergistic partners. The dense neighborhood feature of the network could be used to predict synergism, as has been shown in previous studies [47].

The further investigation of the expression pattern of connected miRNA pairs by calculating their correlation coefficients supported the above hypothesis. It was found that 69% of miRNA pairs had positive co-expression values. This result indicated that most miRNA pairs with synergistic regulations tend to be co-expressed in the developmental and aging process of the human brain. We concluded that the similar expression tendency might ensure synergistic regulations among multiple miRNAs.

In addition, we analyzed the topological properties of the miRNA-miRNA synergistic network between age-related miRNAs and non age-related miRNAs. The average degree and clustering coefficient of age-related miRNAs were much higher, and the average shortest path was much shorter than those of non age-related miRNAs (Table 1). These results suggested that age-related miRNAs tend to be more important and have more synergism within the context of the entire network.

**Table 1:** The topological properties of age-related miRNAs and non age-related miRNAs

	**Mean of AverShortPath**	**Mean of degree**	**Mean of ClusgCoeff**
Age-related	2.987047	32.92391	0.5999161
Non age-related	3.320577	14.02155	0.4912704
P_value	4.988e-11	<2.2e-16	0.0003065

**Figure 6:**
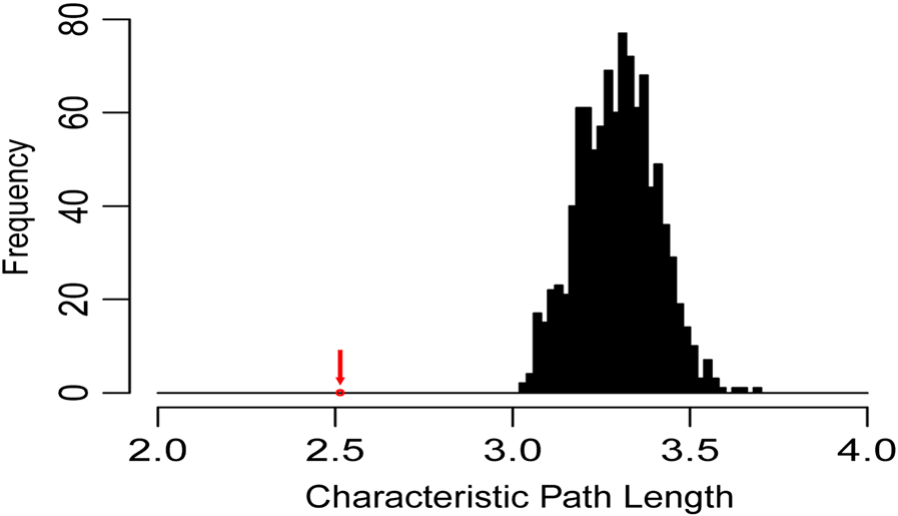
**The characteristic path lengths among age-related miRNAs is shorter than those in randomization tests.** The red arrow represents the characteristic path length of the age-related miRNAs in the actual network.

At last, we calculated the characteristic path length among age-related miRNAs to evaluate the communication efficiency in the network. We randomly selected the same number of miRNAs from the miRNA background set and computed the characteristic path length. This procedure was repeated 1000 times. The characteristic path length of the age-related miRNAs was significantly lower than that derived in the random conditions (Figure 6). The result indicated that age-related miRNAs were closer to each other and communicated quicker than non age-related miRNAs. Thus, it suggested that age-related miRNAs tend to have direct or indirect functional synergy in the developmental and aging process of the human brain.

### Age-related miRNA synergistic modules

Since age-related miRNAs had the higher clustering coefficients and lower characteristic path lengths, and were close to each other, they appeared to implemente regulations as modules. To identify these modules in the synergistic network, we applied Cfinder. All miRNAs in one module were fully connected with each other.

**Figure 7:**
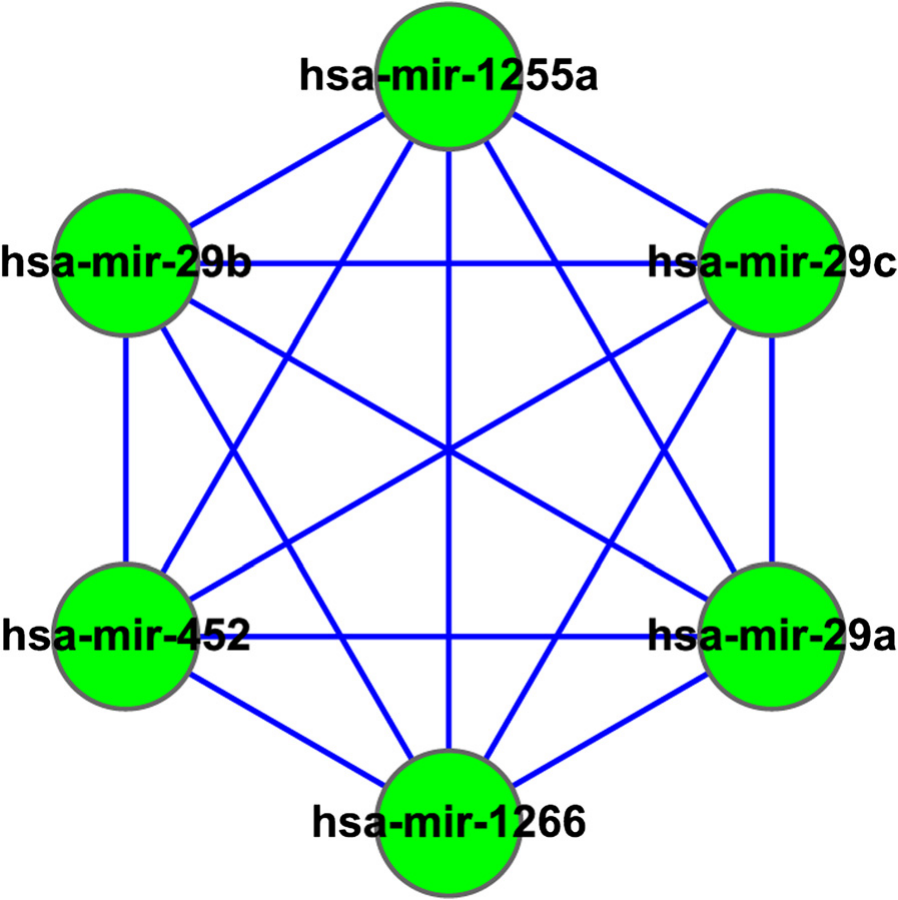
**The miRNA clique 103.** All miRNAs in the clique were fully connected with each other in the miRNA-miRNA synergistic network. All miRNAs in this clique are age-related and related with neuronal development, neurodegenerative diseases and aging-related disorders.

To identify the miRNA synergistic modules related to human brain development and aging, we evaluated the significance of the proportion of age-related miRNAs in each module and tested the correlation of the expression levels of the modules with age by randomly selecting miRNAs as miRNA modules (see the ‘Methods’ section). As a result, 53 age-related miRNA synergistic modules were identified (p < 0.05). The synergistic regulations of modules were then validated by searching target-mRNAs regulated by multiple miRNAs in miRNA synergistic modules. The reliability of regulations between targets and miRNAs was tested by comparing the regulations derived from our work with miRNA-target relationships from ten miRNA target databases. The regulation relationships were reliable if they could be found in at least three databases. For example, miRNA clique 103 (Figure 7) consisted of six miRNAs: hsa-mir-29a, hsa-mir-29b, hsa-mir-1255a, hsa-mir-1266, hsa-mir-452 and hsa-mir-29c. All of them were age-related miRNAs(p_age_pro_ = 0.001). The correlation coefficient between the expression level of the module and age was r = 0.801717991 (p_pearson_ = 0.038).

We found 34 target-mRNAs regulated by at least two miRNAs in clique 103. The regulations between miRNAs and mRNAs were validated by miRNA target databases. This result indicated that miRNAs in clique 103 have synergistic regulations. Furthermore, these 34 target-mRNAs were regulated by hsa-mir-29a, hsa-mir-29b, hsa-mir-29c, hsa-mir-452 and hsa-mir-1266, and were all related to neuronal development, neurodegenerative diseases and aging-related disorders.

Hsa-mir-29a, hsa-mir-29b and hsa-mir-29c are members of the miR-29 family. It is reported that hsa-mir-29a and hsa-mir-29b were down-regulated in the frontal cortex of Alzheimer’s disease (AD), which affected neurodegenerative processes [48]. Moreover, mir-29a, mir-29b and mir-29c were significantly up-regulated, which suggested that they are most likely to play important roles in the developmental and physiological processes during brain development [49]. Hsa-mir-452 was reported to be over-expressed in the WNT signaling associated medulloblastomas [50]. Hsa-mir-1266’s target site spans the rs27072 SNP locus, which was significantly associated with bipolar disorder [51].

The gene NAV3 was synergistically regulated by hsa-mir-29a, hsa-mir-29b, hsa-mir-29c and hsa-mir-452. It was shown that NAV3 expression was enhanced in degenerating pyramidal neurones in the cerebral cortex of AD, while miR-29a was found significantly down-regulated. This observation suggested that under-expression of miR-29a affected neurodegenerative processes by enhancing neuronal NAV3 expression in AD brains [48]. In neuroblastomas, the expression of NAV3 decreased but were up-regulated in nerve cells after brain injury, indicating that NAV3 is involved in neuron growth and regeneration as well as neural tumorigenesis [52]. The gene ARFGEF2 was synergistically regulated by hsa-mir-29a, hsa-mir-29b and hsa-mir-29c. Mutations in ARFGEF2 implicated vesicle trafficking in neural progenitor proliferation and migration in the human cerebral cortex, which was an important regulator of proliferation and migration during human cerebral cortical development [53]. The gene ITPKB was synergistically regulated by hsa-mir-29b and hsa-mir-452, which involved in neuronal calcium dependent signaling, a cellular process related to both AD and aging [54].

## Discussion

In this study, we integrated miRNA and mRNA expression profiles generated from the samples of the human brain across lifespan to construct the miRNA-mRNA regulatory network and the miRNA-miRNA synergistic network. By exploring these two networks, we found that there were significant differences in terms of topological features between age-related miRNAs and non age-related miRNAs. We also found that age-related miRNAs played more important roles than non age-related miRNAs in the developmental and aging process of the human brain. Moreover, the age-related miRNAs tended to work together as modules to affect multiple target mRNAs and have direct or indirect functional synergy in the developmental and aging process of the human brain and in neurodegenerative diseases. Our results were verified by reviewing miRNA target databases and the previous studies.

Most importantly, we revealed the comprehensive regulatory relationships throughout the lifespan. Studying both development and aging simultaneously could revolutionize the methodology in studying structure and function of the human brain and improved our understanding of the physiological regulatory mechanism. Furthermore, we examined the miRNA-mRNA correlations at the whole genome-scale by performing pair-wise spearman correlation coefficient analysis. The relationships based on miRNA target databases can obtain direct regulation between miRNAs and mRNAs, while merely measuring target gene expression may not be sufficient to understand the regulatory effects of miRNAs. As a result, correlation analysis could reveal associations between miRNAs and their target genes as well as non-target genes. Obviously, the synergism of miRNA was implied by the evidence that most mRNAs were co-regulated by over two miRNAs in the miRNA-mRNA network. We obtained the reliable synergism of miRNAs at a system level in the developmental and aging process of the human brain. We considered not only the co-regulation of target genes but also GO function enrichment of co-regulated targets when we constructed the miRNA-miRNA synergistic network, because miRNAs are synergistic in complex diseases and physiological processes, and regulate genes with the same or similar functions.

Our study throws a new light on miRNAs in the developmental/aging system. Also, this work can be extended to study other human tissues if the data is available.

Studying the complex regulatory network between miRNAs and their target genes and the synergism of miRNAs provided more comprehensive understanding of the molecular basis of miRNA functions at a system-wide level.

There are some limitations in our study. First, the miRNA-mRNA correlations are based on pair-wise correlation coefficient analysis. Although we examined the miRNA-mRNA correlations at the whole genome-scale, the identified regulatory correlations might contain false positives. Second, with the limited knowledge of regulation between miRNAs and the developmental and aging process of the human brain, we were unable to complete biological evidences for age-related miRNA synergistic modules. Third, the sample size of the expression profiles was too small. We hope that more comprehensive data could be obtained in the future. Despite these limitations, our study still provides a new insight into the regulation of miRNAs in the developmental and aging process of the human brain.

## Conclusions

In conclusion, age-related miRNAs play more important roles than non age-related miRNAs in the developmental and aging process of the human brain. The age-related miRNAs have synergy effect, and tend to work together as modules.

## Competing interests

The authors declare that they have no competing interests.

## Authors’ contribution

Conceived and designed the experiments: LC. Performed the experiments: WL, WL. Analyzed the data: WL, WL, XQ. Wrote the paper: WL, LC, WL, WH, BL, BC, JJ. Wrote the program code used in the analysis: WL. Contributed reagents/materials/analysis tools: YH, CF, XJ, YZ, JL. All authors read and approved the final manuscript.

## Pre-publication history

The pre-publication history for this paper can be accessed here:

http://www.biomedcentral.com/1755-8794/6/55/prepub
